# Expression of Salivary miR-203a-3p Was Related with Oral Health-Related Quality of Life in Healthy Volunteers

**DOI:** 10.3390/ijms18061263

**Published:** 2017-06-13

**Authors:** Terumasa Kobayashi, Takaaki Tomofuji, Tatsuya Machida, Toshiki Yoneda, Daisuke Ekuni, Tetsuji Azuma, Takayuki Maruyama, Akiko Hirose, Manabu Morita

**Affiliations:** 1 Department of Preventive Dentistry, Okayama University Graduate School of Medicine, Dentistry and Pharmaceutical Sciences, 2-5-1 Shikata-cho, Kita-ku, Okayama 700-8558, Japan; de421015@s.okayama-u.ac.jp (T.K.); de17046@s.okadai.jp (T.M.); de17057@s.okadai.jp (T.Y.); dekuni7@md.okayama-u.ac.jp (D.E.); tetsuji@md.okayama-u.ac.jp (T.A.); mmorita@md.okayama-u.ac.jp (M.M.); 2 Department of Community Oral Health, Asahi University of Dentistry, 1851-1 Hozumi, Mizuho, Gifu 501-0296, Japan; akikohi@dent.asahi-u.ac.jp; 3 Center of Innovative Clinical Medicine, Okayama University Hospital, Okayama 700-8558, Japan; t-maru@md.okayama-u.ac.jp

**Keywords:** biomarkers, microRNA, saliva, oral health-related quality of life

## Abstract

Oral health-related quality of life (OHRQoL) is a multidimensional construct that involves subjective evaluation of an individual’s oral health. Although it is difficult to evaluate OHRQoL biologically, recently, it has been reported that circulating microRNAs (miRNAs) in several body fluids could reflect various health conditions. The aim of this pilot study was to investigate whether salivary miRNAs expression differs according to OHRQoL in healthy volunteers. Forty-six volunteers (median age, 23.0 years) were recruited, and their OHRQoL was assessed using the Japanese version of the Oral Health Impact Profile (OHIP-J). Then, we compared salivary microRNA profiles of the high-OHRQoL group (≤25th percentile score of OHIP-J) and the low-OHRQoL group (≥75th percentile score of OHIP-J) using the polymerase chain reaction (PCR) array and the quantitative real-time PCR. There were no significant differences between the two groups in terms of oral health status. In the PCR array, miR-203a-3p and miR-30b-5p were significantly more expressed in the low-OHRQoL group (*p* < 0.05). Quantitative real-time PCR assay also showed that miR-203a-3p was more highly expressed in the low-OHRQoL group than in the high-OHRQoL group (*p* < 0.05). These observations suggest that expression of salivary miR-203a-3p was related with OHRQoL in healthy volunteers.

## 1. Introduction

Quality of life (QoL) is defined as a person’s perception of his or her position in life. Health contributes to QoL, and health-related QoL (HRQoL) usually focuses on the domains of health status including physical health, mental health, social and role function, and pain and discomfort [1]. Oral health-related quality of life (OHRQoL) reflects total subjective evaluation of an individual’s oral health status, expectations and satisfaction with care, and functional and emotional well-being [2].

Several self-rated questionnaires have been developed to evaluate OHRQoL subjectively [3]. The Oral Health Impact Profile (OHIP) is one of the most commonly used questionnaires used to measure OHRQoL [4]. This instrument contains seven dimensions and is based on Locker’s conceptual model of oral health [5]. The dimensions consisted of functional limitations, physical pain, psychological discomfort, physical disability, psychological disability, social disability and handicap.

However, it is difficult to evaluate QoL objectively. In recent years, it has been reported that biomarkers, such as interleukin-4RA rs 1805010 [6], insulin-like growth factor-1 [7], and N-terminal propeptide of type III procollagen [8], were associated with HRQoL. Although it is conceivable that the use of biomarkers might help to estimate HRQoL objectively, few studies have explored biomarkers for OHRQoL.

MicroRNAs (miRNAs) constitute a recently discovered class of non-coding RNAs that play crucial roles in the regulation of gene expression. Circulating miRNAs in several body fluids (e.g., saliva and serum) have been reported as potential biomarkers of various physiological phenomena. Change in miRNA expression could be associated with changes in pain [9], mental status [10], and social status [11], as well as with oral diseases [12]. Therefore, we hypothesized that some miRNAs in saliva could reflect OHRQoL. The aim of this pilot study was to investigate whether salivary miRNAs expression differs according to OHRQoL in healthy volunteers.

## 2. Results

There were no significant differences in age, gender, number of present teeth, number of decayed, missed, or filled (DMF) teeth, malocclusion, Oral Hygiene Index-simplified (OHI-S), and presence of probing pocket depth (PPD) ≥4 mm between the high- and low-OHRQoL groups (Table 1). None of the volunteers were aware of subjective symptoms of temporomandibular disorders (TMD), oral pain and stomatitis.

**Figure 1:**
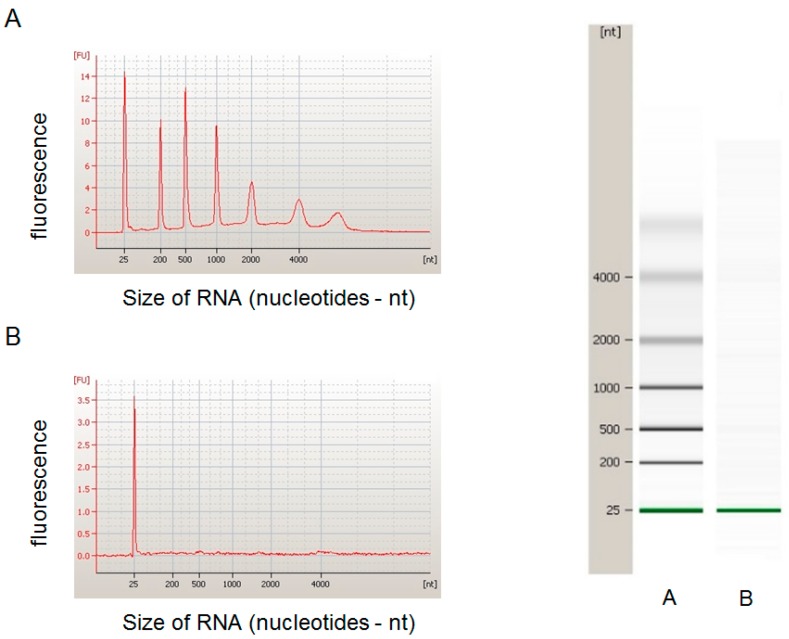
Electropherogram and gel-like image of RNA sample extracted from salivary exosome. *Y*-axis: fluorescence intensity in fluorescence units (FU), *X*-axis: nucleotides (nt). (**A**) RNA ladder; (**B**) An extracted RNA sample.

All RNA from exosome samples had peaks around 25 nucleotides (Figure 1). In polymerase chain reaction (PCR) array analyses, only two miRNAs (miR-203a-3p and miR-30b-5p) were significantly upregulated in the low-OHRQoL group (the common prefix “hsa-” in the miRNA identifications (IDs) were omitted) (Table 2). No miRNA was significantly downregulated.

**Figure 2:**
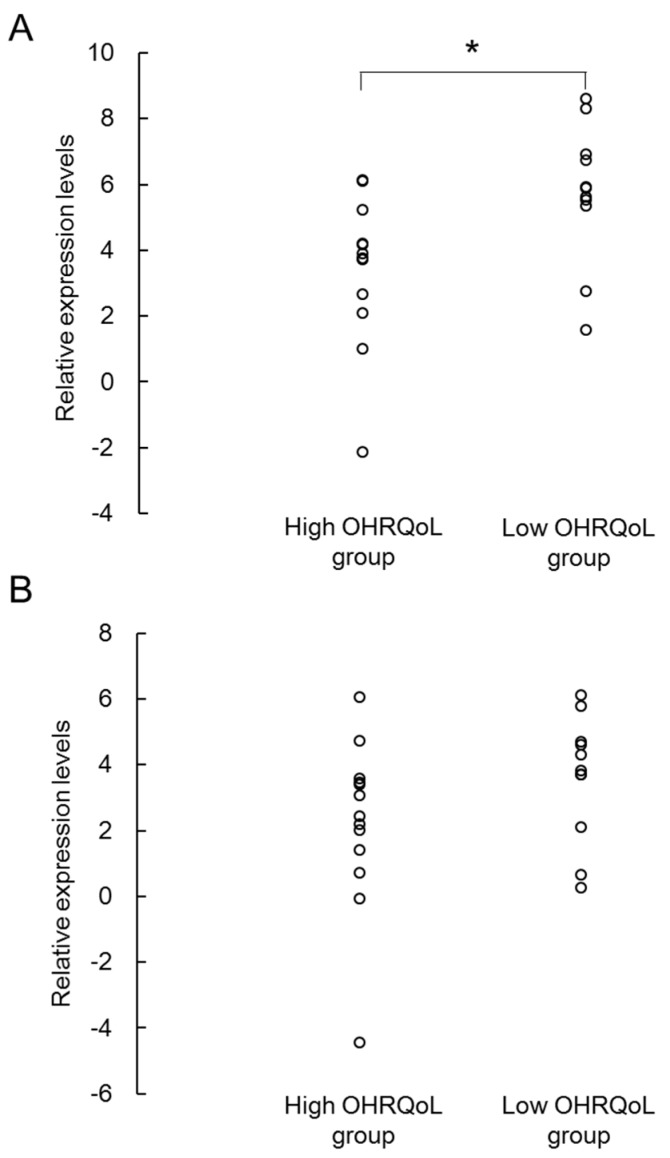
Differential relative expression levels of miR-203a-3p (**A**) and miR-30b-5p (**B**) obtained in the high- and low- oral health-related quality of life (OHRQoL) group. Circle plots represent relative expression rates of each sample. * *p* < 0.05, using the Mann–Whitney *U* test.

In the real-time PCR assays, the low-OHRQoL group induced higher expression rate of salivary miR-203a-3p compared to the high-OHRQoL group (*p* < 0.05) (Figure 2). There was no significant difference in salivary miR-30b-5p between the two groups.

The pathway analysis shows that there was an association between target genes of miR-203a-3p and signaling pathway, including sulfur metabolism, mitogen-activated protein kinase (MAPK), and thyroid hormone (Table 3).

## 3. Discussion

To the best of our knowledge, this is the first study to compare salivary miRNAs profiles of high- and low-OHRQoL groups. At first, we compared the 84 miRNAs expression levels between high- and low-OHRQoL groups. The results showed that two miRNAs (miR-203a-3p and miR-30b-5p) were significantly more expressed in the low-OHRQoL group higher than in the high-OHRQoL group. The quantitative real-time PCR assays confirmed that miR-203a-3p was more highly expressed in the low-OHRQoL group than in the high-OHRQoL group. The results indicate that expression of miR-203a-3p in saliva could reflect OHRQoL.

OHRQoL is related to oral health status [2]. However, in our observations, there was no significant difference in oral health status (number of present teeth, number of DMF teeth, malocclusion, OHI-S, and presence of PPD ≥ 4 mm) between the high- and low-OHRQoL groups. Therefore, the differences in miR-203a-3p between the two OHRQoL groups did not reflect the differences in oral health status. Not only oral health status but also psychological and neuronal factors can affect OHRQoL [2]. It is suggested that miR-203a-3p expression might reflect the status underlying psychological and neuronal factors in the present population. This is consistent with a previous report, which shows a correlation between miR-203a-3p expression and neuron-related protein expression in familial dysautonomia patients [13].

Some patients appeal for a pain without a clinical problem. There are also observed oral inflammation in the patients who feel strange in the mouth. From these clinical experiences, we made the present study hypothesis. However, there were no significant differences in subjective pain and inflammatory conditions between the high- and low-OHRQoL groups in our observations. It is known that OHRQoL was associated with self-related oral health in university students [14]. Self-related oral health may also have an influence on salivary miRNAs expression.

In our analyses, target genes of miR-203a-3p were related to the MAPK pathway, which is involved in pain [15] and inflammation [16]. These suggest that miR-203a-3p can regulate gene expression related with pain and inflammation. Not only does the expression of has-miR-203a-3p in saliva reflect low OHRQoL, but also it may affect the OHRQoL through pain and inflammation at a subclinical level.

Saliva is an oral fluid that is easily accessible by a non-invasive method [17]. Salivary miRNAs were shown to be highly stable because exosomes or protein complexes could protect the salivary miRNAs [18]. Salivary miRNAs were examined as potential biomarkers for oral diseases and systemic diseases [17]. The present study could widen the application range of salivary miRNAs from disease-oriented biomarkers to health-oriented biomarkers such as QoL.

The relationships between circulating biomarkers and HRQoL have been discussed in several articles. The joint subclinical elevation of C-reactive protein and interleukin-6 in plasma was associated with lower HRQoL in the general population [19]. HRQoL scores were strongly related to N-terminal propeptide of type III procollagen in the serum of pulmonary arterial hypertension patients [8]. Elevated baseline serum troponin T levels were independently associated with deterioration in the physical domains of HRQoL in hemodialysis patients [20]. On the other hand, there are few studies discussing relationships between circulating miRNAs and HRQoL. This study emphasizes that not only circulating protein but also circulating miRNAs are candidate biomarkers for HRQoL.

In this study, the mean total OHIP-J score (except for the additional items in the Japanese version) was 16.5. In contrast, it was reported that the mean total OHIP-J score (except for the additional Japanese items, question number 41, and three denture-related items) at 20–29 years of age was 28.9 [21]. These findings indicate that the OHRQoL of the volunteers in this study was better than that of the general population. In addition, because the cut-off level of the OHRQoL has not been yet known, the volunteers with OHIP ≤ 25 percentile were defined as a high-OHRQoL group in this study. However, all the volunteers were Okayama University dental students, suggesting that most of them had high-OHRQoL. These would limit the ability to extrapolate our findings to the general population. Therefore, we should recruit normal patients in the further studies.

Due to the circadian rhythm of the saliva, samples are usually collected in the morning. In this way, a comparison can be made with the other studies. However, in this study, because the students attended the lectures in the morning during the experimental period, we collected saliva in the afternoon. This is a limitation of our study.

The present study had the other limitations. First, we focused on 84 miRNAs related to inflammation and neuropathic pain. However, miRNAs other than those associated with inflammation and neuropathic pain could also be candidate biomarkers for OHRQoL; Second, we performed PCR array analyses using pooled samples but not individual samples; Third, this was a cross-sectional study. Investigating the longitudinal relationship between miRNAs expression and OHRQoL will improve the reliability of this study concept. Further studies are needed to clarify these points.

## 4. Materials and Methods

### 4.1. Study Volunteers and Selection of High- and Low-Oral Health-Related Quality of Life (OHRQoL) Groups

This study was approved by the Ethics Committee of Okayama University Hospital (approval number: 1602-008, 23 February 2016). From April 2016 to June 2016, all 5th-year dental school students at Okayama University (*n* = 46, median age, 23.0 years) participated in this study. None of the volunteers were aware of oral and systemic pain. Moreover, there were no current smokers among the volunteers. Forty-four students did not receive any dental treatment, while two students underwent orthodontic treatment during the experimental period. After obtaining informed consent, volunteers who fulfilled the study requirements were enrolled. This report complies with the Strengthening the reporting of observational studies in epidemiology (STROBE) guidelines for observational studies [22].

In this study, the Japanese version of the OHIP (OHIP-J54) was used to assess OHRQoL [23]. The original OHIP has 49 items, which is related with functional limitation, handicap, physical pain, psychological discomfort, psychological disability, physical disability and social disability. The OHIP-J54 contains five additional items (biting cheeks, difficulty in swallowing, embarrassing joint noises, dry mouth, and poor food texture). The volunteers were asked how frequently they had experienced the impact regarding each question (0, never; 1, hardly ever; 2, occasionally; 3, fairly often; 4, very often). A high total score indicates low OHRQoL. In the present population (*n* = 46), the median (25th, 75th percentile) OHIP-J score was 13.5 (7.0, 20.0). Since no literature is available regarding the cut-off of OHRQoL, volunteers with OHIP score ≤ 25 percentile (OHIP score ≤ 7.0) and OHIP score ≥ 75 percentile (OHIP score ≥ 20.0) were selected in the high-OHRQoL group (*n* = 13) and low-OHRQoL group (*n* = 12), respectively.

### 4.2. Oral Examination

One dentist (Terusama Kobayashi) examined the oral health status of the volunteers. The DMF teeth score (dental caries experience) in the mouth was recorded according to World Health Organization criteria [24]. All teeth were selected for periodontal examination. A Community Periodontal Index probe (Yello Digital Marketing (YDM), Tokyo, Japan) was used to measure PPD at six sites (mesio-buccal, mid-buccal, disto-buccal, disto-lingual, mid-lingual and mesio-lingual) per tooth. The level of dental plaque and calculus was assessed using the OHI-S [25].

### 4.3. Interview

In our previous study, OHRQoL was associated with subjective symptoms of TMD, oral pain and stomatitis in our university students [14]. Therefore, we interviewed students about prevalence of subjective symptoms of TMD, oral pain and stomatitis.

### 4.4. Saliva Collection

At least 1 mL of unstimulated whole saliva was collected as reported previously, with slight modification [26]. Saliva was collected in the afternoon (3:00–4:00 p.m.). The collected saliva was stored at −80 °C until use [27].

### 4.5. Exosome Isolation and RNA Extraction

Total exosome isolation reagent (Invitrogen, Carlsbad, CA, USA) was utilized to isolate exosomes from saliva samples (0.5–1.0 mL) [26]. Total exosome RNA and protein isolation kits (Invitrogen) were used to extract total RNA from exosome samples. To assess the RNA quality, we used an Agilent 2100 bioanalyzer (Agilent Technologies, Carlsbad, CA, USA) and an Agilent RNA 6000 Pico Kit (Agilent Technologies).

### 4.6. PCR Array Analyses and Data Analysis

We conducted miRNA PCR array analyses in triplicate. The Neuropathic & Inflammatory miScript miRNAs PCR Array (SA Biosciences, Frederick, MD, USA), which contains 84 miRNA assays, was used (Table 4). This array included snoRNA/snRNA (SNORD61, SNORD68, SNORD72, SNORD95, SNORD96A and RNU6-6p) for candidate housekeeping small RNA. We used the miScript II RT kit (SA Biosciences) for reverse transcription.

An online tool (SABiosciences; http://pcrdataanalysis.sabiosciences.com/mirna) was used for the data analysis. The ΔΔ*C*_t_ method was used for the relative quantification of miRNAs. The data for the two groups was normalized by the arithmetic mean of several snoRNA/snRNA expressions. The cut-off of threshold cycle (*C*_t_) for the normalization was set at 35 according to the manufacturer’s protocol.

### 4.7. Quantitative Real-Time Polymerase Chain Reaction (PCR)

Quantitative real-time PCR analyses were performed on a Mx3000P Real-time QPCR System (Agilent Technologies) according to the inventoried TaqMan microRNA Assays (Life Technologies, Carlsbad, CA, USA). We analyzed data with a threshold cycle (*C*_t_) value < 50, and calculated the relative expression rates of each miRNA to U6 snRNA (internal control miRNA) using the 2^−ΔΔ*C*t^ method [28].

### 4.8. Bioinformatic Analysis

Bioinformatic analysis was performed using the DIANA-miRPath v3.0 online tool [29].

### 4.9. Statistical Analysis

We compared the high- and low-OHRQoL groups. Student’s *t*-test or the Mann–Whitney *U* test was used to detect differences in the expression levels between the high- and low-OHRQoL groups. Values of *p* < 0.05 were considered to indicate statistical significance.

## 5. Conclusions

Expression of salivary miR-203a-3p was higher in the low-OHRQoL group than in the high-OHRQoL group in healthy volunteers. Salivary miR-203a-3p may serve as a biomarker for OHRQoL.

## Figures and Tables

**Table 1:** Characteristics of high-OHRQoL group and low-OHRQoL group.

Variables	Categories	High OHRQoL Group (*n* = 13)	Low OHRQoL Group (*n* = 12)	*p* Value
		Median or *n*		
Age (years)		23.0 (22.0, 25.5) ^a^	23.0 (22.3, 24.0)	0.650 *
Gender	Male	4 (30.8) ^b^	4 (33.3)	1.000 ^†^
Number of present teeth		28.0 (25.5, 28.5)	28.0 (28.0, 31.8)	0.320 *
Number of DMF teeth		2.0 (0.0, 6.5)	2.0 (0.0, 3.0)	0.650 *
Dental caries-free	Yes	9 (69.2)	9 (75.0)	1.000 ^†^
Malocclusion	Yes	1 (7.7)	3 (25.0)	0.322 ^†^
OHI-S		0.0 (0.0, 1.0)	0.0 (0.0, 1.0)	0.650 *
Presence of PPD ≥ 4 mm	Yes	2 (15.4)	4 (33.3)	0.378 ^†^

*n*: Number, DMF teeth: Decayed, missed, or filled teeth, OHI-S: Oral Hygiene Index-simplified, PPD: Probing pocket depth, OHRQoL: Oral health-related quality of life. ^a^ Median (25 percentile, 75 percentile), ^b^ *n*(%). * Mann–Whitney *U* test, ^†^ Fischer’s exact test.

**Table 2:** The fold change (low-OHRQoL group/high-OHRQoL group) of miRNAs.

miRNA	Upregulated Fold Change	*p* Value
miR-20a-5p	15.67	0.087
miR-16-5p	13.52	0.194
miR-143-3p	12.35	0.209
miR-142-3p	11.63	0.087
miR-29a-3p	10.68	0.104
miR-20b-5p	8.11	0.096
miR-203a-3p	8	<0.001 *
miR-142-5p	7.09	0.313
miR-106b-5p	6.95	0.212
miR-451a	6.81	0.152
miR-30d-5p	5.88	0.06
miR-17-5p	5.55	0.253
miR-93-5p	5.29	0.242
miR-365b-3p	3.82	0.126
miR-30b-5p	3.79	0.017 *
miR-92a-3p	3.65	0.282
miR-221-3p	3.6	0.36
miR-132-3p	3.15	0.071
miR-15b-5p	3.1	0.294
miR-99a-5p	3.02	0.276
miR-145-5p	2.93	0.359
miR-126-3p	2.87	0.166
miR-103a-3p	2.78	0.26
miR-23b-3p	2.65	0.232
miR-125b-5p	2.56	0.263
miR-128-3p	2.49	0.336
miR-30a-3p	2.49	0.221
miR-30c-5p	2.43	0.179
miR-199b-3p	2.39	0.602
miR-25-3p	2.33	0.335
miR-92b-3p	2.31	0.104
miR-34a-5p	2.08	0.215
miR-133b	0.31	0.189
miR-34c-5p	0.4	0.105
miR-133a-3p	0.45	0.172
miR-10a-5p	0.47	0.344
miR-127-3p	0.47	0.479
miR-155-5p	0.48	0.092

The *p* values are calculated based on a Student’s *t*-test. * *p* < 0.05.

**Table 3:** Pathway analysis of target genes for miR-203a-3p, which was expressed higher in the low-OHRQoL group than in the high-OHRQoL group.

Pathway	Target Genes	*p* Value
Sulfur metabolism (hsa00920)	*PAPSS2*	0.008
MAPK signaling pathway (hsa04010)	*NTRK2*, *CRK*, *RAP1A*, *MAP3K1*, *TAOK1*, *PPM1A*, *PRKCB*, *MEF2C*, *DUSP5*, *MAP3K5*	0.041
Thyroid hormone signaling pathway (hsa04919)	*THRB*, *PRKCB*, *PLCE1*	0.041

We used DIANA-miRPath v3. 0. (http://www.microrna.gr/miRPathv3). MAPK: mitogen-activated protein kinase.

**Table 4:** List of 84 miRNA used in this study.

**Upregulated by Pain:**
Upregulated by Contusive Spinal Cord Injury: miR-1, miR-145-5p, miR-146a-5p, miR-146b-5p, miR-152-3p, miR-15b-5p, miR-17-5p, miR-199a-3p, miR-203a, miR-206, miR-20a-5p, miR-20b-5p, miR-214-3p, miR-21-5p, miR-223-3p, miR-30a-3p, miR-31-5p, miR-374b-5p, miR-378a-3p, miR-92a-3p, miR-92b-3p, miR-98-5p.
Upregulated by Ischemia-Reperfusion Injury: miR-132-5p, miR-133a-3p, miR-204-3p, miR-323a-3p, miR-365b-3p, miR-369-5p, miR-376b-5p, miR-505-5p, miR-665.
**Downregulated by Pain:**
Downregulated by Contusive Spinal Cord Injury: miR-129-2-3p, miR-137, miR-138-5p, miR-219a-2-3p, miR-219a-5p, miR-30b-5p, miR-30c-5p, miR-30d-5p, miR-323a-3p, miR-338-5p, miR-34a-5p, miR-379-3p, miR-495-3p, miR-543.
Downregulated by Inflammatory Pain: miR-10a-5p, miR-124-3p, miR-134-5p, miR-183-5p, miR-29a-3p, miR-98-5p, miR-99a-5p.
Downregulated by Ischemia-Reperfusion Injury: miR-146a-5p, miR-199a-3p, miR-210-3p.
**Regulated by Pain:**
Regulated by Contusive Spinal Cord Injury: miR-100-5p, miR-103a-3p, miR-107, miR-124-3p, miR-127-3p, miR-128-3p, miR-133a-3p, miR-133b, miR-154-5p, miR-181a-5p, miR-451a, miR-487b-3p, miR-99a-5p.
Complex Regional Pain Syndrome: miR-106b-5p, miR-126-3p, miR-130b-3p, miR-132-3p, miR-142-5p, miR-155-5p, miR-16-5p, miR-181a-5p, miR-20a-5p, miR-20b-5p, miR-221-3p, miR-25-3p, miR-93-5p.
Responsive to Antiinflammatories: miR-142-3p, miR-146b-5p, miR-203a, miR-208a-3p, miR-21-5p, miR-219a-2-3p, miR-219a-5p, miR-302d-3p.
Responsive to Neuroprotection: miR-199a-3p, miR-210-3p, miR-219a-2-3p, miR-323a-3p, miR-324-5p, miR-331-5p, miR-365b-3p, miR-369-5p, miR-376b-5p, miR-7-5p.
Apoptosis: miR-1, miR-106b-5p, miR-125b-5p, miR-128-3p, miR-133a-3p, miR-133b, miR-134-5p, miR-143-3p, miR-145-5p, miR-146a-5p, miR-15b-5p, miR-16-5p, miR-17-5p, miR-181a-5p, miR-183-5p, miR-199a-3p, miR-203a, miR-206, miR-20a-5p, miR-210-3p, miR-214-3p, miR-21-5p, miR-221-3p, miR-25-3p, miR-29a-3p, miR-30b-5p, miR-30c-5p, miR-30d-5p, miR-31-5p, miR-323a-3p, miR-34a-5p, miR-34c-5p, miR-365b-3p, miR-378a-3p, miR-451a, miR-7-5p, miR-92a-3p, miR-98-5p.
Inflammation: miR-106b-5p, miR-125b-5p, miR-127-3p, miR-128-3p, miR-130b-3p, miR-145-5p, miR-146a-5p, miR-15b-5p, miR-16-5p, miR-17-5p, miR-181a-5p, miR-199a-3p, miR-20a-5p, miR-20b-5p, miR-21-5p, miR-210-3p, miR-23b-3p, miR-29a-3p, miR-30b-5p, miR-30c-5p, miR-30d-5p, miR-323a-3p, miR-34a-5p, miR-34c-5p, miR-365b-3p, miR-543, miR-93-5p, miR-98-5p, miR-99a-5p.
Associated with Neuropathic Pain in Specific Cells:
Amygdala: miR-182-5p, miR-34c-5p.
Dorsal Root Ganglia Nociceptor: miR-103a-3p, miR-133a-3p, miR-134-5p, miR-143-3p, miR-20a-5p, miR-20b-5p, miR-21-5p, miR-7-5p.
Hippocampus: miR-125b-5p, miR-132-5p.
Immune Cells: miR-124-3p, miR-132-5p.
Insular Cortex: miR-133b.
Prefrontal Cortex: miR-181a-5p, miR-223-3p.
Spinal Dorsal Horn: miR-103a-3p, miR-124-3p, miR-134-5p, miR-23b-3p.
Other miRNA Involved in Pain: miR-339-5p, miR-96-5p.

## Abbreviations

QoL	Quality of life
HRQoL	Health-related QoL
OHRQoL	Oral health-related QoL
OHIP	Oral health impact profile
MiRNAs	MicroRNAs
MAPK	Mitogen-activated protein kinase
DMF teeth	Decayed, missed, or filled teeth
OHI-S	Oral hygiene index-simplified
PPD	Probing pocket depth
TMD	Temporomandibular disorders
